# Detection and quantification of membrane-damaging antimicrobials using pHluorin2-based bacterial biosensors

**DOI:** 10.3389/fmicb.2026.1856943

**Published:** 2026-07-09

**Authors:** Julia Zaraza, Niklas Fante, Alexander Grünberger, Alexander Schretzmeier, Jonas Stohr, Oliver Goldbeck, Christian U. Riedel, Christian K. Desiderato

**Affiliations:** 1 Microbial Biotechnology, Institute of Evolutionary Ecology and Conservation Genomics, University of Ulm, Ulm, Germany; 2 Institute of Process Engineering in Life Sciences: Microsystems in Bioprocess Engineering, Karlsruhe Institute of Technology, Karlsruhe, Germany

**Keywords:** activity assay, bacteriocins, biosensor, fluorescence, membrane damage, pH

## Abstract

Bacteriocins are ribosomally synthesized antimicrobial peptides of bacterial origin. Many known bacteriocins kill susceptible bacteria by interacting with cell surface receptors and disrupting membrane integrity, leading to the collapse of pH homeostasis. The fluorescent protein pHluorin2 is a GFP-derivative characterized by two distinct excitation peaks at 400 and 475 nm that change in relative fluorescence intensities in a ratiometric manner in response to changes in pH. Bacteria expressing pHluorin2 show fluorescence profiles characteristic for their intracellular pH. When placed in buffer at a pH that is different from the intracellular pH by ±1–2 units, these bacteria are able to maintain pH homeostasis. However, when treated with compounds that disrupt membrane integrity such as many bacteriocins, the intracellular pH rapidly changes to the pH of the buffer resulting in a change in the fluorescence profile of pHluorin2 within seconds. This change can be assessed with any method that is able to detect fluorescence intensities at the two excitation maxima of the protein. Hence, pHluorin2-expressing bacteria can be used to measure the activity of membrane-damaging antimicrobials by orders of magnitude faster than with conventional, growth-dependent assays. The methods presented here describe construction and use of live biosensor bacteria that express pHluorin2 for investigation of membrane-damaging compounds such as bacteriocins. Applications of these biosensors include characterization, screening, and quantification by various methods comprising spectrophotometry in microtiter plate readers, microscopy, and flow cytometry. Additionally, the protocol introduces a procedure for preparation of ready-to-use assay plates with freeze-dried pHluorin2 biosensor bacteria.

## 1 Introduction

Increasing antibiotic resistance of pathogenic microorganisms remains one of the most urgent problems for global human health (World Health Organization, 2014, 2025). Antimicrobial peptides are a conserved mechanism of host defense found in all domains of life and are discussed as alternatives to conventional antibiotics (Cotter et al., 2013; Mookherjee et al., 2020; Dijksteel et al., 2021; Dad et al., 2025; Ngashangva et al., 2026). Bacteriocins are a subset of antimicrobial peptides produced by bacteria primarily to compete for resources in the same natural habitat by interfering with the growth of other bacteria (Riley and Wertz, 2002; Heilbronner et al., 2021). Although various mechanisms of action are known for different bacteriocins, the most common mechanism involves formation of pores in the cytoplasmic membrane with subsequent disruption of membrane potential and cell integrity (Cotter et al., 2013; Sugrue et al., 2024).

**Figure 1:**
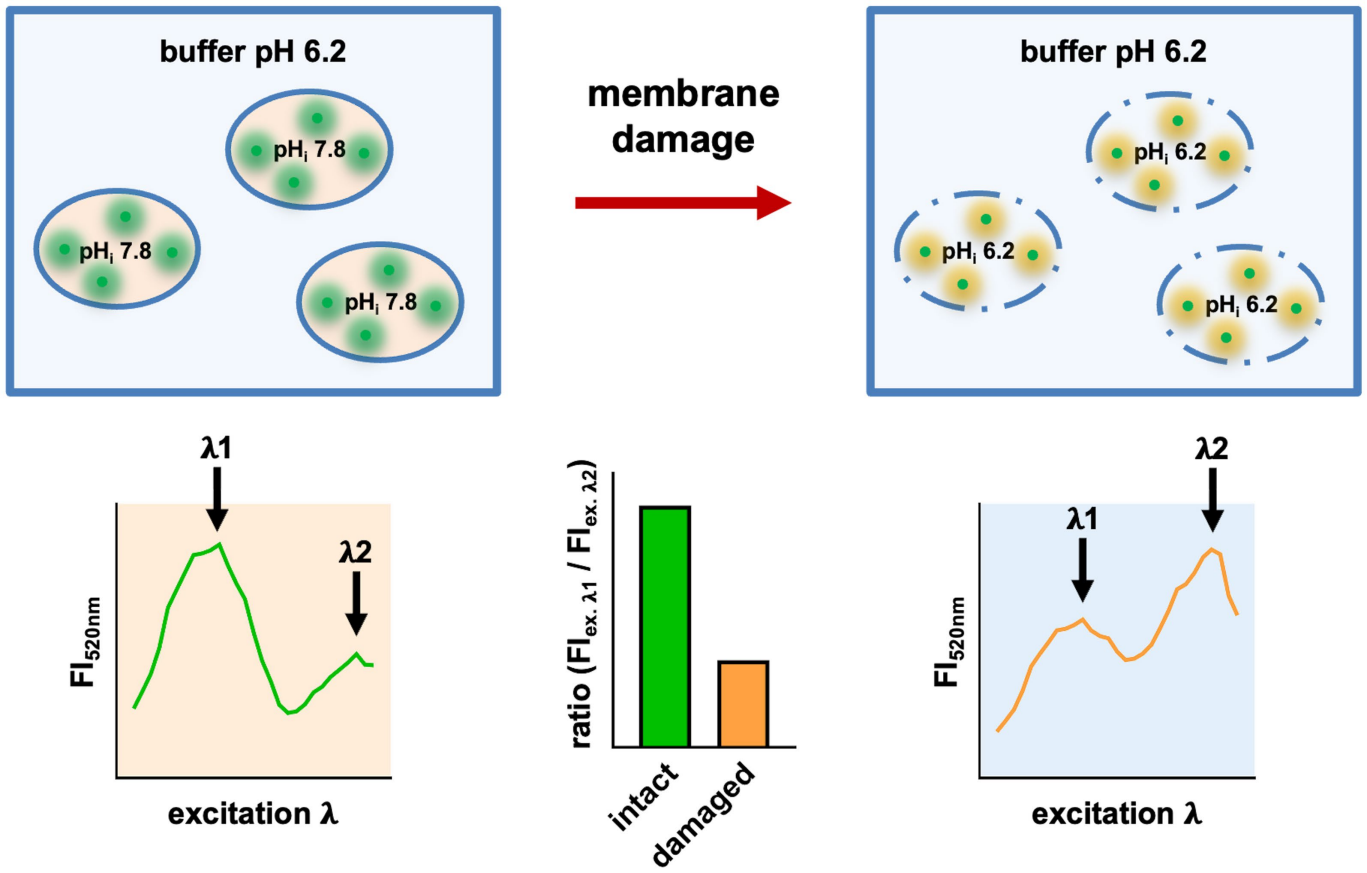
Schematic representation of the pH-dependent fluorescent properties of pHluorin2 biosensor bacteria to assess membrane integrity or damage. Bacteria with intact cytoplasmic membrane are able to maintain pH homeostasis (e.g., pH_i_ = 7.8 for *L. monocytogenes*) even when placed in a slightly acidic buffer (pH 6.2, left side, green spectrum and bar). These biosensor bacteria show a characteristic excitation profile of FIs at 520 nm reflecting their pH_i_ and a higher FI after excitation at maximum λ1 (400 nm, left graph). When exposed to compounds that disrupt membrane integrity, pH_i_ immediately changes to the extracellular pH of the assay buffer (left side, orange spectrum and bar). Consequently, biosensor bacteria show a higher FI after excitation at maximum λ2 (480 nm, right graph). This change can be measured rapidly by determining the ratio of FIs at 520 nm after excitation at λ1 and λ2 (ratio (FI_ex. λ1_ / FI_ex. λ2_); bar chart, middle).

We developed live fluorescent *Listeria monocytogenes* biosensors that allow fast, easy, and cost-efficient detection of compounds with membrane-damaging activity including bacteriocins and other substances (Crauwels et al., 2018). These biosensors express pH-dependent derivatives of the green fluorescent protein that is characterized by two distinct excitation peaks at 400 and 480 nm spectrum, whose relative fluorescence intensities (FIs) at 520 nm show a ratiometric shift in response to changes in pH (Miesenböck et al., 1998; Mahon, 2011). In other words, the ratio of fluorescence intensities after excitation at the two peaks changes with pH. When the cytoplasmic membrane is intact, bacteria are able to maintain pH homeostasis even when placed in a buffer with pH different by ±1–2 units from the intracellular pH (pH_i_). Thus, biosensor bacteria expressing pHluorin show a characteristic ratio of FIs at 520 nm when excited at 400 and 480 nm, reflecting their pH_i_. However, when exposed to compounds that disrupt membrane integrity, pH_i_ immediately changes to the extracellular pH of the assay buffer. This shift can be used to detect the presence of membrane-damaging compounds by changes in the ratio of FIs after excitation at 400 and 480 nm within minutes or even seconds (Figure 1).

After successful establishment of this method for investigation of bacteriocins, live bacteria biosensors were further improved for *Listeria* spp. by expression of pHluorin2, a derivative of pHluorin with enhanced FI (Reich et al., 2022; Mahon, 2011). More recently, the system was adopted for generation of pHluorin2 biosensors of other bacterial genera including strains of *Bacillus cereus*, *Staphylococcus epidermidis*, *Staphylococcus aureus*, *Lactococcus lactis*, *Corynebacterium glutamicum*, and *Escherichia coli* (Desiderato et al., 2022; Pashou et al., 2022; Otto et al., 2024). Here, we describe a set of generalized protocols for generation of bacterial pHluorin2 biosensors and their application for identification and characterization of membrane-active compounds, which may also be of use to investigate other compounds with membrane-damaging activity.

## 2 Materials and equipment

Reagents, microbiological media, chemicals, materials, and instruments used for the described methods are listed in Supplementary Data S1, Tables S1–S3 unless already described in the sections blow. *Listeria innocua* LMG2785/pNZ-pHin2*^Lm^* was published previously (Reich et al., 2022) and harbors pNZ-pHin2*^Lm^* for constitutive expression of pHluorin2. *E. coli* DH5α/pNZ-pHin2*^Lm^* was used to propagate and isolate pNZ-pHin2*^Lm^* for generation of *Lactococcus lactis* IL1403/pNZ-pHin2*^Lm^* (Desiderato et al., 2022), *E. coli* MG1655/pNZ-pHin2*^Lm^* (Otto et al., 2024), and *Staphylococcus carnosus* TM300/pNZ-pHin2*^Lm^* (this study). For cloning of further pHluorin2-plasmids (see Protocol 3.1 below), any suitable cloning host (e.g., one of the many commercially available *E. coli* strains) may be used. *Escherichia coli* strains were cultivated in standard LB medium at 37 °C. *L. innocua* and *S. carnosus* strains were grown in BHI broth at 37 °C and *L. lactis* strains in GM17 medium at 30 °C. For selection, chloramphenicol was added at appropriate concentrations depending on the organism.

## 3 Methods and protocols

### 3.1 Construction of pHluorin2 biosensor bacteria

This protocol describes generation of a biosensor strain of *L. innocua* LMG2785 as previously published (Reich et al., 2022). This strain constitutively expresses pHluorin2 using the plasmid backbone of pNZ44, a broad range expression vector for Gram-positive bacteria that also contains an origin of replication functional in *E. coli* (McGrath et al., 2001). Consequently, pNZ44-based plasmids were successfully used for generation of was also used for generation of a wide range of bacteria including *L. monocytogenes* EGDe, *Bacillus cereus* DSM 31, *S. epidermidis* RP62-a, methicillin-resistant *Staphylococcus aureus* Rosenbach, *L. lactis* IL1403, and *E. coli* MG1655 (Desiderato et al., 2022; Otto et al., 2024).

1. Cut pNZ44 (McGrath et al., 2001) using restriction enzymes *Bgl*II and *Pst*I (FastDigest enzymes, Thermo Fisher Scientific). This results in the linearized pNZ44 backbone and a second fragment harboring the p44 promoter, which is subsequently separated by agarose gel electrophoresis. The band containing the linearized pNZ44 backbone is excised from the agarose gel and purified using QIAquick Gel Extraction Kit (Qiagen).
2. Amplify the strong, constitutive P_help_ promoter by PCR using Q5 polymerase (New England Biolabs), pPL2*lux*P_help_ (Riedel et al., 2007) as template, primers P_help__fw and P_help__rv (sequences see Supplementary Data 1, Table S4), and the standard PCR protocol recommended by the supplier of the polymerase^1^ with 61.9 °C annealing temperature and 15 s elongation time.
3. A synthetic DNA fragment with a *pHluorin2* gene codon-optimized for *L. monocytogenes* (see Supplementary Data 1, Table S4) is obtained from a commercial service provider (Eurofins Genomics) and amplified by PCR using Q5 polymerase, primers pHin2LM_fw and pHin2LM_rv (sequences see Supplementary Data 1, Table S4), and the standard PCR protocol with 58.6 °C annealing temperature and 45 s elongation time.
4. Assemble vector backbone and PCR products in a single isothermal reaction as described elsewhere (Gibson et al., 2009) using the sequences at the end of the PCR products homologous to the ends of the pNZ44 backbone added by PCR with the primers (see Steps 2 and 3). Transform the reaction mix into chemocompetent *E. coli* DH5α (Thermo Scientific) and select transformants on LB agar plates containing 30 μg mL^−1^ chloramphenicol (Sigma-Aldrich). Isolate plasmid pNZ-pHin2*^Lm^* of positive clones and verify relevant parts of this new plasmid (i.e., *pHluorin2* gene in correct orientation) by restriction analysis and Sanger sequencing (primers: P_help__fw and pHin2LM_rv) by a commercial service provider (Microsynth Seqlab) to confirm correct cloning of was obtained pNZ-pHin2*^Lm^*.
5. Transform correct plasmids into *L. innocua* LMG2785 by electroporation using a previously described protocol (Monk et al., 2008) and select positive clones on brain heart infusion (BHI) agar plates containing 10 μg mL^−1^ chloramphenicol.

In case pHluorin2 expression and/or FI is insufficient for detection in a microtiter plate reader or by flow cytometer and/or the replicon pNZ44 replicon is not functional in a target organism, cloning can be performed using suitable expression vector, codon-optimized, synthetic *pHluorin2* genes and/or highly active, constitutive promoters suitable for the target host. This has been demonstrated for, e.g., a pHluorin2 biosensor of *C. glutamicum* ATCC13032 expressing a codon-optimized *pHluorin2* gene under control of the strong, constitutive P*_tuf_* promoter (Weixler et al., 2022). Cloning can be adopted to any strategy employing classical restriction/ligation procedures or more advanced methods. Optimized transformation protocols may have to be used depending on the host organisms.

### 3.2 Microtiter plate assay

This protocol described procedures optimized for the *L. innocua*/pNZ-pHin2*^Lm^* and standard conditions for cultivation are BHI agar or broth containing 10 μg mL^−1^ chloramphenicol, incubation at 37 °C under aerobic conditions (e.g., on a rotary shaker at 130–200 rpm depending on the instrument). Conditions may need to be adapted to biosensors of other bacteria.

1. Inoculate a single colony of biosensor bacteria from a freshly grown agar plate into 5 mL of liquid medium with appropriate antibiotics and incubate for 6 h at 37 °C with aeration.
2. Transfer the 5 mL pre-culture into 50 mL BHI with 10 μg mL^−1^ chloramphenicol (or an antibiotic appropriate for the biosensor) in a baffled shake flask and incubate overnight at 37 °C with aeration.
3. After overnight growth, biosensor bacteria are harvested by centrifugation at 4,500 × g for 10 min at room temperature and washed once with 50 mL of saline (0.9% (w/v) NaCl in H_2_O).
4. After determination of OD at 600 nm (OD_600_) of the bacterial suspension, of biosensors are centrifuged again at 4,500 × g for 10 min at room temperature and resuspended in optimized *Listeria* minimal buffer (LMBo: 200 mM 2-(*N*-morpholino)ethanesulfonic acid, 4.82 mM K_2_HPO_4_, 11.55 mM Na_2_HPO_4_, 1.7 mM MgSO_4_, 0.6 mg mL^−1^ (NH_4_)_2_SO_4_, 55 mM glucose, pH 6.2, components dissolved in demineralized H_2_O, no autoclaving, buffer filter-sterilized prior to use). The volume of LMBo is calculated to obtain a final OD_600_ of 3, which is then confirmed by measurement of an appropriately diluted sample.
5. Biosensors are transferred into a 50 mL falcon tube wrapped with tin foil and stored for no longer than 30 min before proceeding with Step 6, or use in other protocols (see below).
6. Distribute samples (max. 100 μL) as well as suitable controls (e.g., neg. Control: 100 μL LMBo; pos. Controls: 100 μL LMBo containing 10 μg mL^−1^ of ultrapure nisin Z (Handary) into individual wells of a black 96-well MTP). For quantitative analysis of activity, prepare serial two-fold dilutions by dispensing 100 μL of LMBo into all wells for dilutions. Then, add 100 μL of a sample to the first well of a row/column, mix well by pipetting and serially transfer 100 μL across rows/columns using a multichannel pipette (if available).
  NOTE: Low pH and presence of organic acids in supernatants of, e.g., lactic acid bacteria may compromise the signal and produce false positive signals. Thus, when testing plain supernatants of potential bacteriocin producers or purified bacteriocins in solvents, it is recommended to test culture supernatants of a related non-producer, sterile medium and/or solvents for background fluorescence or assay interference.
7. Dispense 100 μL of biosensor suspension (prepared in Steps 1–5) into each well of the MTP (using a multichannel pipette, if available), wrap the MTP in tin foil, and incubate at room temperature for 30 min.
8. Measure fluorescence emission at 520 nm in each well, either across spectrum of excitation wavelengths (350–490 nm) or, for more rapid measurements, after excitation at 400 and 480 nm using an MTP reader. The ratio of FIs after excitation at 400 and 480 nm can be directly used as a proxy for the pH of the milieu surrounding the pHluorin2 protein (i.e., intracellular pH) and can be calibrated against crude extracts of the biosensor prepared in buffer at different pH.
  NOTE: A third measurement of FI after excitation at 440 nm can be used as a quality control (Otto et al., 2024). A non-linear regression calculated with a second order polynomial fit (f(x) = ax^2^ + bx + c) using the three FIs after excitation at 400, 440, and 480 nm has a positive ax^2^ component (parabola open to the top) indicate correct measurements. On the other hand, negative ax^2^ component of the fit (parabola open to the bottom) indicates compromised signals due to, e.g., denatured pHluorin2 protein.

Since pHluorin2 is a GFP-derivative that requires oxygen for maturation and high fluorescence, results are best for biosensor cultivated under aerobic conditions, ideally in shake flasks. For anaerobes, maturation of fluorescent proteins may occur after cultivation by incubation in the presence of oxygen (Grimm et al., 2014). However, expression and functionality of pHluorin2 have not been demonstrated in anaerobic bacteria so far.

### 3.3 Ready-to-use MTPs with freeze-dried of pHluorin2 biosensors

This protocol provides ready-to-use MTPs with biosensors that can be stored at −20 °C for longer periods of time. It is optional but may be helpful for high throughput screenings or if variability between experiments needs to be minimized. This protocol has been developed and tested for pHluorin2 biosensors of *L. innocua* LMG2785, *S. carnosus* TM300, and *L. lactis* IL1403. The procedure may be applicable to other related bacteria but may need optimization at various points depending on the storage conditions of the biosensors (e.g., buffer systems and cryoprotectants such as mannitol or BSA instead of sucrose).

**Figure 2:**
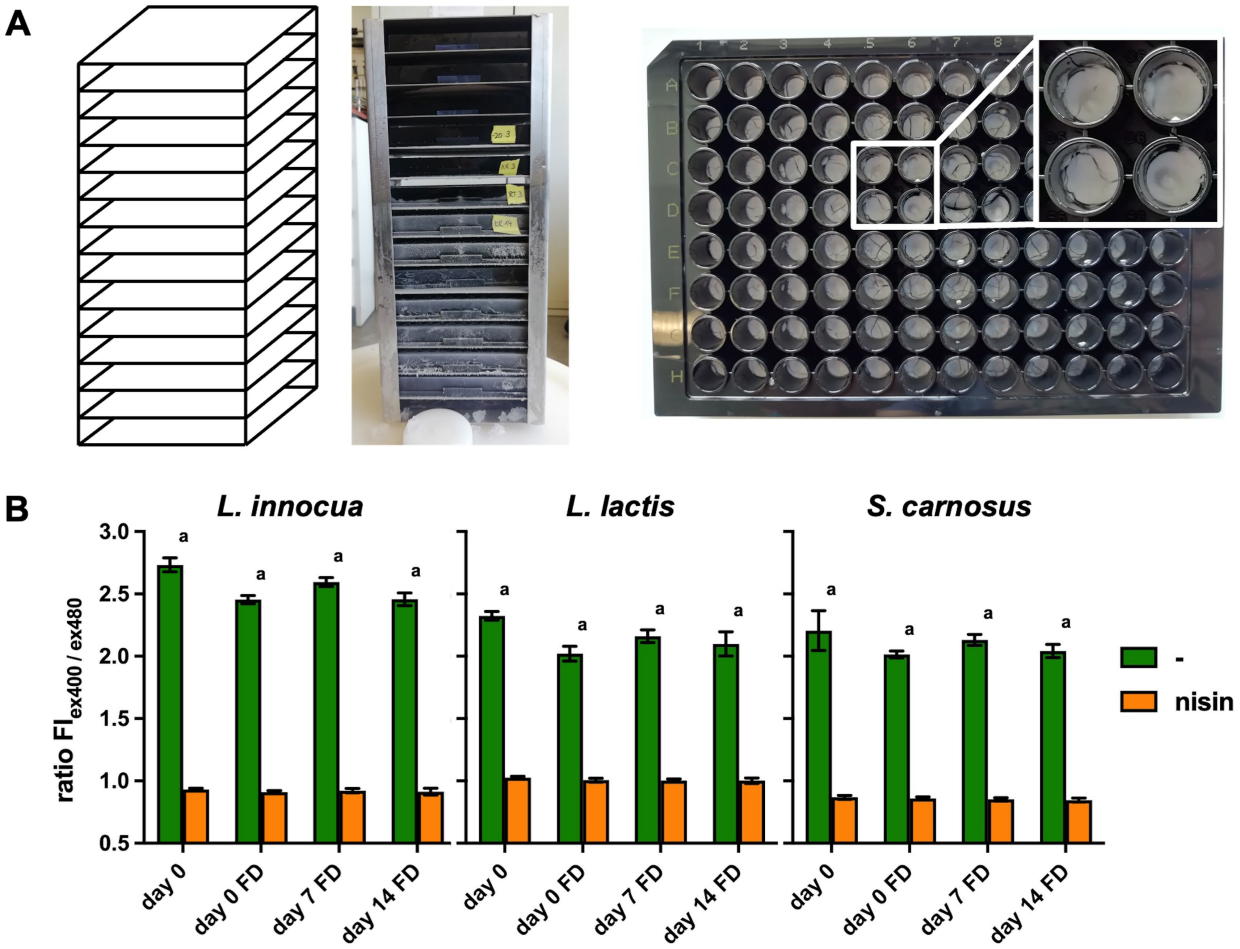
**(A)** Schematic drawing (left) and image (middle) of the metal rack used to freeze pHluorin2 biosensor bacteria in LMBoS buffer at −80 °C prior to lyophilization. After lyophilization, freeze-dried biosensor bacteria in cryoprotectant form a stable and homogenous layer on the bottom of the MTP wells (right). **(B)** FI ratios (ratio FI_ex400 / ex480_) of intact (green bars) and nisin-permeabilized (orange bars) biosensors in ready-to-use MTPs before (day 0) and after freeze-drying (day 0 FD) as well as after storage for 7 (day 7 FD) and 14 (day 14 FD) days of storage at −20 °C demonstrate stability of cryopreserved pHluorin2 biosensor bacteria. All values are mean ± standard deviation of *n* = 3 independent cultivations of biosensors. Statistical analysis was performed by Student’s t-test (unpaired, two-tailed) comparing treated vs. untreated biosensors at each timepoint. Statistically significant differences to untreated biosensor bacteria are indicated by letters (a: *p* < 0.001).

1. Prepare the biosensor cells as described in Steps 1–5 of Protocol 3.2. However, biosensors were resuspended in LMBoS buffer (LMBo containing 10 mg mL^−1^ sucrose, pH 6.2) instead of LMBo. The additional sucrose in the LMBoS buffer prevents cell damage during freeze-drying.
2. Distribute 100 μL biosensor suspension to each well of an MTP, wrap MTPs with tin foil and incubate at −80 °C for 24 h.
3. The next day, remove tin foil and place MTPs in a metal rack (see Figure 2A) and incubate for 2 h at −80 °C while cooling down freeze-dryer to −53 °C.
  NOTE: The metal rack is essential for two critical steps during preparation of the ready-to-use MTPs. Precooled metal rack accelerates freezing of biosensors at −80 °C. Also, it keeps MTPs chilled and prevents cryopreserved bacteria from defreezing during transfer from −80 °C to the freeze-dryer.
4. Place metal rack with precooled MTPs in the freeze-dryer as quick as possible to avoid thawing of the frozen cell suspension.
5. Ramp down vacuum in freeze-dryer slowly and evenly to ~ 0.1 mbar and incubate MTPs for 24 h in the freeze-dryer at −53 °C ice condenser temperature and under vacuum.
6. The next day, release the vacuum by slowly opening freeze dryer valve.
  NOTE: If the vacuum is released abruptly, the freeze-dried cell pellets might pop out of the MTP wells.
7. Cover each MTP with Breathe-Easy® sealing membrane and tin foil and store at −20 °C until further use.
8. Before performing the pHluorin2 assay with ready-to-use MTPs, add 100 μL demineralized water to each well of the MTP and incubate for 10 min to rehydrate the freeze-dried cell pellets.
9. In the meantime, prepare samples in a separate MTP as described in Step 6 of Protocol 3.2.
10. Transfer the samples quickly from the separate MTP to the biosensor-containing MTP, wrap the MTP in tin foil and incubate at room temperature for 1 h.
11. Measure and evaluate results of pHluorin2 assay as described in Step 8 of Protocol 3.2.

### 3.4 Analysis of pHluorin2 biosensors in microfluidics by fluorescence microscopy

This protocol describes single-cell analysis of pHluorin2 biosensors with high spatio-temporal resolution in microfluidic systems using fluorescence microscopy. Analysis by fluorescence microscopy in microfluidic systems is optional and provides information on potential cell-to-cell heterogeneity of the biosensor cells as well as time resolved data of the biosensor signal. The procedures described are optimized for *L. innocua* LMG2785/pNZ-pHin2^Lm^ biosensors and standard conditions for cultivation (Fante et al., 2024).

1. Fabricate and prepare the microfluidic chip.
  NOTE: Perform all open-handling steps (preparation, mixing, curing, bonding) in a clean bench/laminar flow hood whenever possible to minimize particle contamination. This reduces the risk of clogged channels, damaged structures, imperfect bonding, and imaging artifacts during experiments.
  - Prepare the wafer and carefully remove any traces of dust or other residues prior to use. If required, rinse with 2-propanol and dry thoroughly using compressed air.
  - Prepare two-component polydimethylsiloxane (PDMS) by mixing 10 vol. of base and 1 vol. of curing agent in a suitable vessel. The required total amount depends on the size and number of microfluidic chips to be fabricated. As reference, approximately 2 g of PDMS are needed for chip a chip with a size of 15 × 20 × 3 mm. Stir thoroughly with a spatula until the mixture is homogeneous.
  - Pour PDMS mix onto the wafer until all structures are fully covered sufficiently with PDMS.
  - Place the wafer in a desiccator and degas under reduced pressure to remove trapped air bubbles. Repeat as needed until no bubbles remain within or above the structures.
  - Cure PDMS by baking at 80 °C for 2 h. Remove the wafer from the oven and allow it to cool to room temperature.
  - Cut around the patterned area using a scalpel. Avoid scratching the microstructures and leave a small margin around the structures to maintain integrity during subsequent steps.
  - Demold by carefully peeling the cured PDMS mold from the wafer without deforming or tearing the structures. If needed, use a clean scalpel or spatula to gently lift an edge and initiate separation.
  - To punch the inlet and outlet holes using a 0.75 mm biopsy punch, place the PDMS mold on a clean surface (ideally use a PDMS block as base) and punch in one slow, steady motion. Gentle twisting the puncher can improve quality of cuts.
  - To fix PDMS mold on a glass slide for microscopy, clean both mold and glass slide by removing dust and other particles with compressed air. Rinse both the PDMS mold and the glass substrate with 2-propanol, then dry with compressed air. Ensure 2-propanol is fully evaporated before proceeding and specifically check inlet/outlet holes where liquid may remain.
  - Place the PDMS mold and glass slide in a plasma generator with the bonding surfaces facing upwards for plasma activation and bonding. Activate under low-pressure oxygen plasma (0.8 mbar) for 24 s at 90 W. After plasma activation, vent the plasma chamber to atmospheric pressure and remove the PDMS mold and glass substrate. Immediately bring the activated surfaces into contact to achieve covalent bonding and create functional microfluidic chips.
2. Prepare the biosensor cells as described in Steps 1–5 of Protocol 3.2.
3. Set up the required microfluidic cultivation periphery (tubing, connectors, pumps/pressure controller, media; for setup see Fante et al., 2024).
4. Start and set up the fluorescence microscope. If time-lapse imaging is performed and an environmental/incubator chamber is available, start it and allow it to equilibrate before beginning image acquisition.
5. Mount and fix the microfluidic chip on the microscope stage. Focus on the chip structures and adjust microscope settings.
6. Take a sample of biosensor bacteria and inoculate the microfluidic chip.
7. Connect the pressurized pump system containing the prepared media (e.g., medium with and without bacteriocin) to the inlets. Start perfusion with medium without bacteriocin to establish ideal growth conditions and to start on-chip cultivation of biosensors.
8. Perfuse the chip with growth medium until the desired population size is reached.
9. Select regions of interest (ROIs) in the microscopy software for time-lapse imaging. Ensure the chamber area is homogeneously populated with scattered single cells to (i) minimize supply limitations/nisin depletion effects and (ii) distinguish single-cell fluorescence heterogeneity from proliferation-related effects.
10. For temporally resolved measurements, define the acquisition interval (based on the required resolution – here 1 min) and start imaging.
11. Switch from growth medium to medium containing bacteriocin by changing the active inlet flow. When analyzing repeated exposure to bacteriocin with recovery phases without bacteriocin, switching between media can be performed at predefined time points.
12. After completing the experiment, export and save all acquired image data from the microscope control/acquisition software.
13. Analyze the data using suitable image-processing software, e.g., Fiji (Schindelin et al., 2012).

### 3.5 Analysis of pHluorin2 biosensors by flow cytometry

Analysis of by flow cytometry allows to observe cell-to-cell heterogeneity of pHluorin2 biosensors upon AMP treatment (Fante et al., 2024). When analyzing bacteriocin samples with high background fluorescence (e.g., supernatants of cultures grown in highly complex media), analysis of pHluorin2 biosensor bacteria by flow cytometer may help to reduce background fluorescence (Otto et al., 2024). It is recommended to perform flow cytometry on freshly prepared pHluorin2 biosensor bacteria. Analysis of freeze-dried biosensors from ready-to-use assays plates (see Protocol 3.3) is possible but data is compromised by a larger proportion of non-fluorescent cells and debris.

1. Prepare the biosensor cells and perform pHluorin2 assay like described in Steps 1–7 of Protocol 3.2.
2. During incubation of the pHluorin2 assay, prepare the flow cytometer by cleaning, initializing, calibrating, and initializing the flow cytometer and adjust instrument settings.
3. After preparing the flow cytometer, add 5 μL of biosensor sample (see Step 1) to 50 μL flow cytometer grade PBS.
4. Analyze the diluted sample in the flow cytometer by recording the required parameters (FSC, SSC), fluorescence intensity in the 528/46 channel for both excitation lasers (405 and 488 nm) of at least 10,000 events.
5. Analyze the data by a suitable analysis software for flow cytometry data by setting a gate for bacterial cells based on FSC and SSC, then gate singlet events based on FSC aspect ratio and subsequent dot-plot with FI 528/46 (ex. 405 nm) over FI 528/46 (ex. 488 nm).
  NOTE: If gating for singlet events based on the FSC aspect ratio is not possible, omit this step and use the bacteria population for the next step.

## 4 Results and discussion

**Figure 3:**
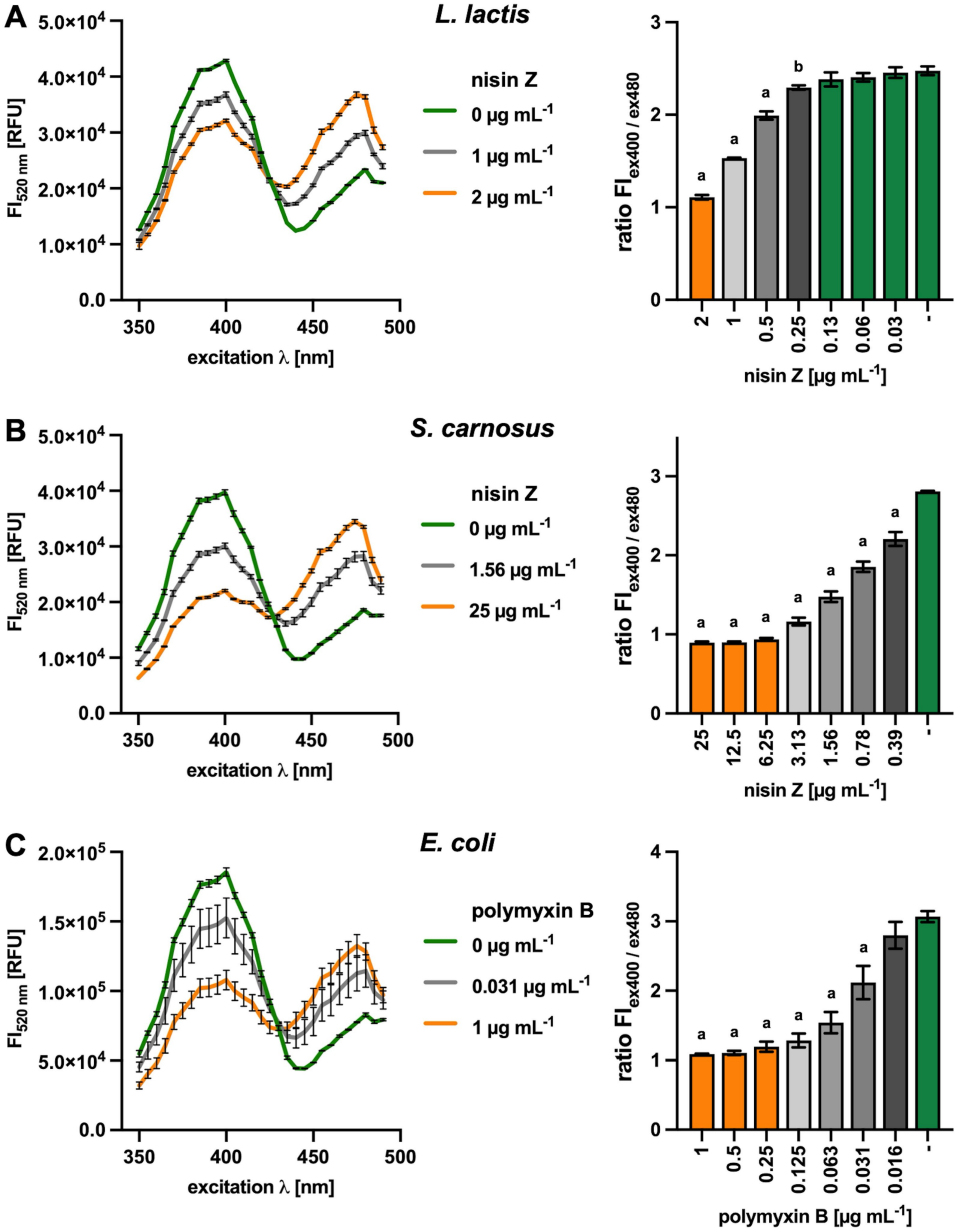
Characterization of biosensor bacteria *L. lactis* IL1403/pNZ-pHin2*^Lm^* **(A)**, *S. carnosus* TM300/pNZ-pHin2*^Lm^* **(B)**, and *E. coli* MG1655/pNZ-pHin2*^Lm^* **(C)**. After treatment with nisin Z at the indicated concentrations, fluorescence intensity at 520 nm (FI) were recorded as relative fluorescence units (RFU) after across a spectrum for excitation wavelengths (350–490 nm, left panels) or at the two maxima (400 and 480 nm). Using FIs at the two excitation maxima FI ratios (ratio FI_ex400 / ex480_) were calculated (right panels). All values are mean ± standard deviation of *n* = 3 independent cultivations of biosensors. Statistical analysis was performed by ANOVA and Dunnett’s multiple comparisons test to calculate *p*-values. Statistically significant differences to untreated biosensor bacteria are indicated by lettes (a: *p* < 0.001; b: *p* < 0.01).

To demonstrate the applicability of Protocol 3.1, *S. carnosus* TM300 was transformed with pNZ-pHin2*^Lm^* using methods for generation and transformation of competent bacteria published elsewhere (Löfblom et al., 2007). *S. carnosus* TM300/pNZ-pHin2*^Lm^* and previously published *L. lactis* IL1403/pNZ-pHin2*^Lm^* and *E. coli* MG1655/pNZ-pHin2*^Lm^* biosensors (Desiderato et al., 2022; Otto et al., 2024) were characterized in an MTP assay as described in Protocol 3.2. Following incubation of these biosensors with different concentrations of commercial ultrapure nisin Z (Handary) or polymyxin B (Sigma-Aldrich) for 30 min at room temperature, FI (emission: 520 nm) at the two excitation maxima (400 and 480 nm) were recorded and FI ratios (FI_400nm / 480nm_) were calculated (Figure 3; left panels).

In line with previous studies, all pHluorin2 biosensors displayed the typical bimodal fluorescence at baseline with high emission FI when excited at 400 nm and a second, somewhat lower peak in FI when excited at 480 nm (Figures 3A,B). Moreover, for the two Gram-positive biosensors *S. carnosus* TM300/pNZ-pHin2*^Lm^* and *L. lactis* IL1403/pNZ-pHin2*^Lm^* relative FI at these two peaks shifted (reduced FI when excited at 400 nm and increased FI when excited at 480 nm) when treated with high (lethal) concentrations of nisin Z. Also, an intermediate spectrum was observed at intermediate nisin concentrations. Similar observations were made when only FI ratios were considered as indicator of the average cell integrity across the population of biosensor bacteria. Ratio of 2–3 (depending on the biosensor) indicates cells with intact pH homeostasis (intracellular pH 7.5–7.8) whereas disruption of membrane integrity results in shift of the intracellular pH to 6.2 (i.e., pH of LMBo) and a drop in the FI ratio to ~ 1. Nisin concentrations required for complete shift of FI ratio differed markedly between the two biosensor bacteria suggesting different levels of tolerance to nisin. Also, intermediate (i.e., sublethal) concentrations resulted in incomplete shift in FI ratio for all biosensors analyzed.

In contrast to the Gram-positive biosensors, only a minor effect was observed when *E. coli* MG1655/pNZ-pHin2*^Lm^* was treated with nisin Z at 50 μg mL^−1^ (data not shown). This is in line with the considerably lower activity of nisin against Gram-negative bacteria, which is, at least partially, due to the limited access to its receptor lipid II caused by impermeability of the outer membrane for nisin (Field et al., 2023). However, the characteristic shift in the excitation spectrum of *E. coli* MG1655/pNZ-pHin2*^Lm^* was observed after treatment with 1 μg mL^−1^ of the pore-forming peptide antibiotic polymyxin B (Figure 3C; left panel). Similar to the Gram-positive biosensors, *E. coli* MG1655/pNZ-pHin2*^Lm^* showed a partial shift of the fluorescence spectrum at an intermediate polymyxin B concentration (0.031 μg mL^−1^) and a gradual decrease in the FI ratios when treated with a increasing concentrations (Figure 3C; right panel). Overall, this demonstrates that Gram-negative biosensors have characteristics and responses to membrane-active compounds comparable to those of Gram-positive biosensors.

To minimize batch-to-batch variations and facilitate high throughput screenings, Protocol 3.3 was developed for preparation of ready-to-use MTPs with cryopreserved biosensor bacteria containing either *L. innocua* LMG2785/pNZ-pHin2*^Lm^*, *L. lactis* IL1403/pNZ-pHin2*^Lm^* or *S. carnosus* TM300/pNZ-pHin2*^Lm^*. Stability of ready-to-use MTPs without loss in signal quality depends on storage temperature but when stored at −20° C the fluorescent properties of all strains and their response to nisin were preserved (Figure 2B). Assays performed with ready-to-use MTPs with all three biosensors provided stable pHluorin2 readouts for at least 2 weeks of storage.

For *L. innocua* LMG2785/pNZ-pHin2*^Lm^*, we also assessed the effect of cryopreservation and storage on biomass and viability of bacteria. To this end, OD_600_ and colony forming units (CFU) were quantified in ready-to-use MTPs immediately prior lyophilization of assay plates and after lyophylization and storage for 14 days (Supplementary Data 1, Figure S5). OD_600_ slightly dropped during storage from 1.51 ± 0.02 to 1.47 ± 0.01 (*p* < 0.05) but no significant decrease in CFU mL^−1^ was observed. As FIs did not change markedly upon lyophylization and storage and FI ratios allowed for a good detection of nisin activity in both cases, the decrease in OD_600_ was considered negligible. Although similar stability of biosensors remains to be confirmed, the stable FI and good detection of nisin activity by *L. lactis* IL1403/pNZ-pHin2*^Lm^* or *S. carnosus* TM300/pNZ-pHin2*^Lm^* indicates that these biosensors are also sufficiently stable in ready-to-use MTPs and during storage.

**Figure 4:**
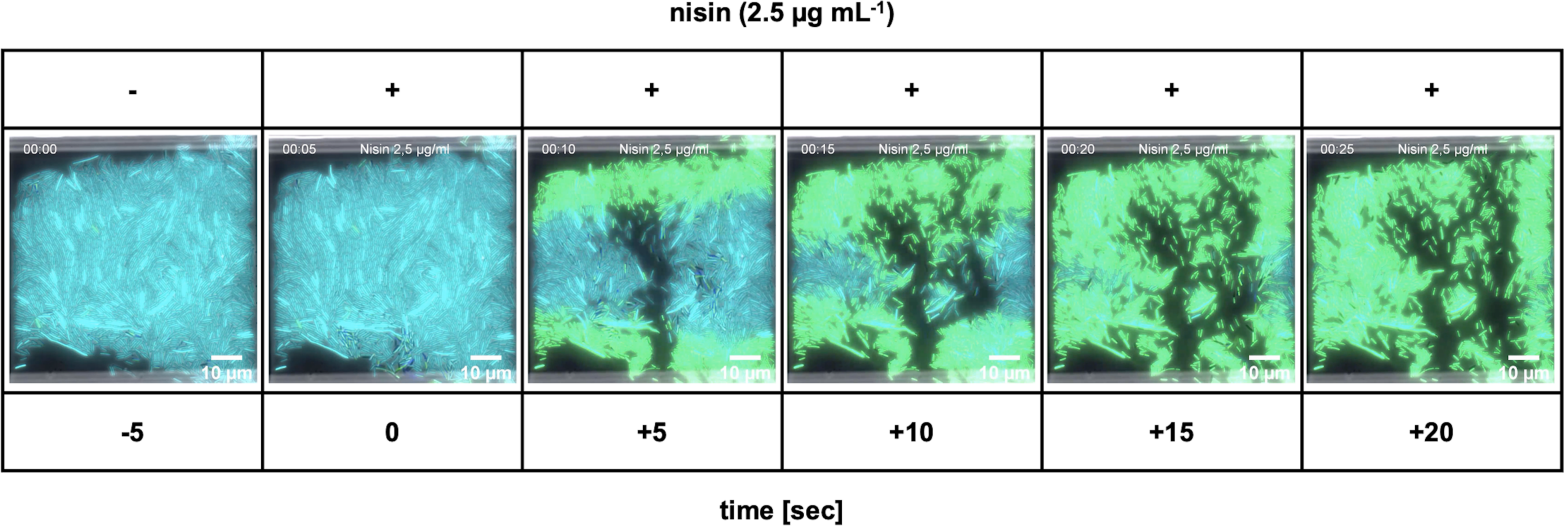
Individual frames of time-lapse fluorescence microscopy of *L. innocua* LMG2785/pNZ-pHin2*^Lm^* exposed to 2.5 μg mL^−1^ of nisin Z. Images were acquired on an Eclipse Ti2 Series fluorescence microscopy (Nikon) with the following settings: objective: 100 × oil immersion; fluorescence channel 1: excitation 390/40 nm, emission 520/35 nm, illumination intensity 10%, exposure time 400 ms; fluorescence channel 2: excitation 472/30 nm, emission 520/35 nm, illumination intensity 10%, exposure time 100 ms. Each frame is an overlay of images acquired in phase contrast mode and the two fluorescent channels in false-color overlay.

In MTP assays, biosensors are usually incubated with bacteriocins for 30–60 min prior to fluorescence measurements. Protocol 3.4 was used to study the temporal dynamics of the response of a defined number of bacteria of *L. innocua* LMG2785/pNZ-pHin2*^Lm^* biosensors exposed to a lethal concentration of nisin on single-cell level in a microfluidic chip by time-lapse fluorescence microscopy (Figure 4; Supplementary Movie S2). This revealed that bacteria at the outer boundaries of the microfluidic chip exposed first to nisin displayed a switch in FI at the two excitation maxima within seconds upon onset of exposure. Moreover, within 15–20 s, almost all biosensor bacteria had undergone this switch indicating complete loss in membrane integrity. Together this demonstrates the fast kinetics of nisin activity and confirms that pHluorin2 biosensors provide a read-out within seconds, which is faster by several orders of magnitude compared to conventional, growth-dependent assays to measure bacteriocin activity.

The shift in pHluorin2 FI ratio of all biosensor tested was concentration dependent and a partial shift of the ratio was observed at bacteriocin concentrations lower than those required for complete killing (Figure 3; Reich et al., 2022; Otto et al., 2024). Similar observations were made for some samples in a screening of supernatants of LAB collections with up to 400 strains (Desiderato et al., 2021; Otto et al., 2024). Intermediate signals may either be derived from homogenous populations of biosensors with partially disturbed membrane integrity or by a mixed population consisting of biosensor bacteria with fully intact or completely disrupted membranes. Fluorescence microscopy of pHluorin2 biosensor bacteria in microfluidic setups may also be used to investigate potential population heterogeneity in response to exposure to bacteriocins on the single-cell level as demonstrated elsewhere (Fante et al., 2024).

**Figure 5:**
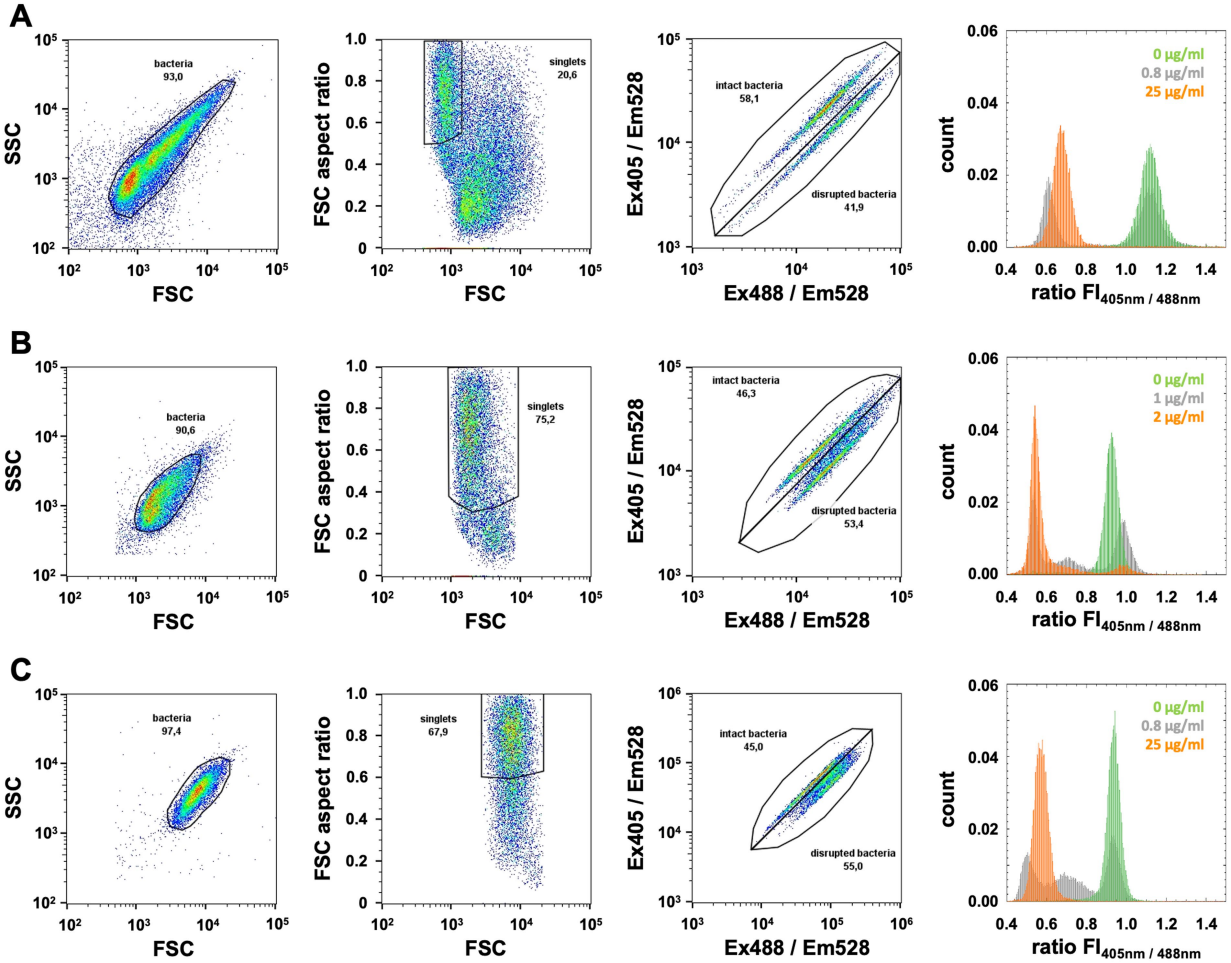
Flow cytometry of *L. innocua* LMG2785/pNZ-pHin2*^Lm^* **(A)**, *L. lactis* IL1403/pNZ-pHin2*^Lm^* **(B)** and *S. carnosus* TM300/pNZ-pHin2*^Lm^* **(C)** biosensors. Bacteria were identified based on their forward and side scatter (FSC, SSC; left panels). To remove bacterial aggregates with ambiguous fluorescent signals, singlet events were gated by plotting FSC aspect ratio over FSC (second panels from the left). Finally, pHluorin2-positive singlets were analyzed for fluorescence intensity (emission wavelength 528 nm) after excitation with the 405 nm (Ex405/Em528) and 488 nm laser (Ex488/Em528; third panels from the left). Dot plots are shown for biosensor treated with sublethal concentrations of ultrapure nisin Z (0.8 μg mL^−1^ for *L. innocua* and *S. carnosus*, 1.0 μg mL^−1^ for *L. lactis*) to obtain two distinct populations. Similar analyses were performed with biosensors prior to nisin-challenge or treated with lethal nisin Z concentrations (25 μg mL^−1^ for *L. innocua* and *S. carnosus*, 2.0 μg mL^−1^ for *L. lactis*). FI ratios were calculated using FIs of individual cells in the Ex405/Em528 and Ex488/Em528 channels exported and values were MATLAB to generate histogram plots (right panels). Data was acquired on an Amnis® CellStream® IS100 device with the following setting: flow speed “slow”; laser power for FSC and SSC: 10%; laser power 405 nm: 35%; laser power 488 nm: 40%. Data of one representative of at least *n* = 3 independent cultivations of each biosensor is shown.

Another method to assess fluorescence properties of bacteria on single-cell level is flow cytometry, which has also be applied to analyze pHluorin2 biosensors (Reich et al., 2022; Fante et al., 2024; Otto et al., 2024). To demonstrate the feasibility of this approach, Protocol 3.5 was used to quantify the effects of different concentrations of nisin on *L. innocua* LMG2785/pNZ-pHin2*^Lm^*, *L. lactis* IL1403/pNZ-pHin2*^Lm^* and *S. carnosus* TM300/pNZ-pHin2*^Lm^* biosensors (Figure 5). This revealed that for all biosensors the vast majority of bacteria (>98%) showed bright fluorescence at 528 nm when excited at either 405 or 488 nm confirming homogenous expression of pHluorin2 by the biosensor cells. Moreover, two predominant populations were observed. These two populations were even more pronounced when exporting fluorescence intensities in the two channels for individual bacterial cell and calculating fluorescence intensity ratios in MATLAB (Figure 5, right panels). One of the two populations was characterized by either a high RFU ratio of ~ 1 representing live, intact bacteria whereas the second population showed a lower RFU ratio of ~ 0.5. Untreated biosensors uniformly consisted of intact, live bacteria and after treatment with high concentrations the entire population shifted to lower RFU-ratios indicating loss in membrane-integrity. At intermediate concentration of nisin both populations were observed. This confirms observations in previous studies and demonstrates that analysis of pHluorin2 biosensor bacteria by flow cytometry allows to detect, discriminate bacteria with intact or damaged membrane on single-cell level. Of note, a third, intermediate population was observed at sublethal nisin Z concentrations for *L. lactis* IL1403/pNZ-pHin2*^Lm^* and *S. carnosus* TM300/pNZ-pHin2*^Lm^* biosensors. We hypothesize that this third population may be related to the tendency of these organisms to from aggregates/chains of two or more cells that contain both intact and disrupted bacteria. Although this needs to be substantiated by further investigation, e.g., by fluorescence microscopy, it coincides with the absence of a clear population of singlet events. Moreover, similar observations were reported previously with a pHluorin2 biosensor of methicillin-resistant *S. aureus* Rosenbach (Otto et al., 2024), a bacterium that is also know to form aggregates.

Most of the pHluorin2 biosensors available so far are Gram-positive bacteria and, in most cases, labeling has been achieved with pNZ-pHin2*^Lm^* harboring a *pHluorin2* gene codon-optimized for *L. monocytogenes*. For the vast majority of biosensors, this provides sufficient FI to allow application of the strains in both MTP assays and flow cytometric analyses. However, levels of FI differ between biosensors. For example, FI of *L. innocua* B486/pNZ-pHin2*^Lm^* is about an order of magnitude higher than the signal of *S. aureus* Rosenbach/pNZ-pHin2*^Lm^* (Otto et al., 2024). As MTPs and flow cytometers differ in sensitivity due to differences in the optical principles for excitation and detection of fluorescence, FI of some biosensors, e.g., *S. aureus* Rosenbach/pNZ-pHin2*^Lm^* and *B. cereus* DSM31/pNZ-pHin2*^Lm^* is insufficient for detection in MTP readers (Table 1). Also, high background fluorescence in samples to be tested (e.g., supernatants of bacteriocin producers cultivated in complex media) may reduce signal:noise ratios and further lower detection limits in MTP assays. In case of *C. glutamicum* ATCC13032, these limitations could be overcome by changing the expression system to a different plasmid backbone (pPBEx2) and a *pHluorin2* gene codon-optimized specifically for the host. Similar approaches may help to improve biosensor with signals currently insufficient for MTP assays. However, in some cases codon-optimization and changing plasmids did not improve biosensors to allow good detection. For example, neither changing the plasmid backbone nor *pHluorin2* codon-optimization has provided suitable biosensors for *Pseudomonas* species (data not shown). Although strains could easily be generated, high background fluorescence in the range of pHluorin2 was observed possibly due to the presence of fluorogenic metabolites such as pyoverdine (Xiao and Kisaalita, 1995).

**Table 1:** Comparison of all pHluorin2-based biosensor bacteria available so far, the plasmid used for labeling, biosafety level (BSL) as well as their suitability for use in the methods described above (MTP, microtiter plate assays; FC, flow cytometry assays; micr., microscopy).

Strain	plasmid	BSL	Method			Ref.
			MTP	FC	micr.*	
*Listeria innocua* B486	pNZ-pHin2*^Lm^*	1	+	+	+	Reich et al. (2022)
*Listeria monocytogenes* EGDe	pNZ-pHin2*^Lm^*	2	+	+	+	Reich et al. (2022)
*Lactococcus lactis* IL1403	pNZ-pHin2*^Lm^*	1	+	+	n.t.	Desiderato et al. (2022)
*Corynebacterium glutamicum* ATCC13032	pPB-pHin2*^Cg^*	1	+	+	n.t.	Weixler et al. (2022)
*Staphylococcus aureus* Rosenbach	pNZ-pHin2*^Lm^*	2	−	+	n.t.	Otto et al. (2024)
*Staphylococcus epidermidis* RP62a	pNZ-pHin2*^Lm^*	2	+	+	n.t.	Otto et al. (2024)
*Staphylococcus carnosus* TM300	pNZ-pHin2*^Lm^*	1	+	+	−	this study
*Bacillus cereus* DSM31	pNZ-pHin2*^Lm^*	2	−	+	n.t.	Otto et al. (2024)
*Escherichia coli* MG1655	pNZ-pHin2*^Lm^*	1	+	+	n.t.	Otto et al. (2024)

^*^ n.t.: not tested.

## 5 Conclusion

The protocols and assays described here have proven useful for high-throughput screening of, e.g., supernatants of large collections of industrial starter cultures or environmental isolates for membrane-damaging activity (Desiderato et al., 2021; Otto et al., 2024). Moreover, the MTP assay may also be used to monitor activity during optimization of (recombinant) productions and complex purification protocols with several precipitation and chromatography steps. A major advantage of pHluorin2 biosensors is the time to detection of a signal. While classical growth-dependent assays used to investigate bacteriocins (e.g., growth inhibition, disk diffusion or spot-on-lawn assays) require 24–48 h to produce results (Balouiri et al., 2016). By contrast, pHluorin2 biosensors provides results within 2 h or less including time for preparation of the biosensors and samples and the assay itself.

Despite their advantages over classical, growth-dependent assays, pHluorin2 biosensors are limited to detection and analysis of antimicrobial compounds that act by damaging the membrane. Nevertheless, these limitations can be overcome by combining pHluorin2 assays with other methods albeit at the cost of extended times for assays and evaluation. Also, MTP assays with pHluorin2 biosensors capture integrated signals across an entire population of cells, biosensors can also be used to detect heterogeneity in response to bacteriocins on single-cell level (Fante et al., 2024) and assess these heterogeneities quantitatively (Otto et al., 2024). Here, pHluorin2 biosensors provide a signal without addition of exogenous enzymes, substrates or dyes required for other methods such as propidium iodide staining or ATP bioluminescence assays (Balouiri et al., 2016).

As a GFP derivative, pHluorin2 requires oxygen for maturation. Although labeling of strict anaerobes such as bifidobacteria with GFP and other fluorescent protein has been demonstrated but requires oxygen (e.g., by incubation on the bench) for maturation. Hence, generation of pHluorin2 biosensors of anaerobes may be possible if these bacteria remain intact and able to maintain membrane homeostasis in the presence of oxygen. This, however, has not been demonstrated so far.

In summary, we provide a collection of protocols for rapid, easy, cost-efficient, and reliable analysis of compounds with membrane-damaging activity in different samples and settings. While pHluorin biosensors do not detect antimicrobial compounds that act by other mechanisms unless combined with other methods. Some of these protocols are particularly suitable for HTS but all of these protocols may be used in mechanistic studies to demonstrate a specific mode of action of an antimicrobial.

## Data Availability

The raw data supporting the conclusions of this article will be made available by the authors, without undue reservation.

## Footnotes

1
https://www.neb.com/en/protocols/pcr-using-q5-high-fidelity-dna-polymerase-m0491
