# Supplementary material for: Detection and quantification of membrane-damaging antimicrobials using pHluorin2-based bacterial biosensors
Source: Front Microbiol. 2026 Jul 9;17:1856943. doi: 10.3389/fmicb.2026.1856943 (PMC13391539; doi:10.3389/fmicb.2026.1856943)
Supplement: Supplementary file 1 [file Data_Sheet_1.pdf]

Supplementary Material

Detection and quantification of membrane-damaging antimicrobials using pHluorin2-based bacterial biosensors

Julia Zaraza[1], Niklas Fante[2], Alexander Grünberger[2], Alexander Schretzmeier[1], Jonas Stohr[1], Oliver Goldbeck[1], Christian U. Riedel[1*], Christian K. Desiderato[1]

[1] Microbial Biotechnology, Institute of Evolutionary Ecology and Conservation Genomics, University of Ulm, Ulm, Germany

[2] Institute of Process Engineering in Life Sciences: Microsystems in Bioprocess Engineering, Karlsruhe Institute of Technology, Karlsruhe, Germany

[*] **Correspondence:** christian.riedel@uni-ulm.de; ORCID: 0000-0001-7134-7085

1.1 Supplementary Tables

Table S1: Microbiological media used for the described methods.

Medium*	Formulation
BHI	BD Difco™ Brain Heart Infusion, 37 g L^{-1}
GM17	NutriSelect® Basic M17 broth, 37 g L^{-1}, 55 mM glucose (added after autoclaving from a filter-sterilized stock solution)
LB	10 g L^{-1} Gibco Bacto™ Tryptone, 5 g L^{-1} Bacto™ Yeast Extract, 10 g L^{-1} NaCl

*: All media were sterilized by autoclaving. For solid media, 16 g L^{-1} BD Bacto™ Agar were added prior to sterilization.

Table S2: Chemicals and material used for the described methods.

Chemicals/Material	Manufacturer/Supplier	Model/Catalog #
Adapter needles for microfluidics	Nordson	7018316
Amnis® CellStream® Calibration Reagent	Cytek®	CS-400104
Breathe-Easy® sealing membrane	Sigma-Aldrich	Z380059
ELISA plate, 96 well, flat base, PS, black, Medium Binding	Sarstedt	82.1581.120
FlowClean Cleaning Agent	Beckman Coulter	C48093
Glass substrate for fabrication of microfluidic chips	Schott	D 263 T eco
Glucose monohydrate	Carl Roth	6780.2
K_2HPO_4	Sigma-Aldrich	P3786-1KG
2-(N-morpholino)ethanesulfonic acid	Carl Roth	4259.5
$MgSO_4 \times 7\ H_2O$	Carl Roth	P027.2
MTP metal rack	Scientific Workshop - University of Ulm	Custom design
Na_2HPO_4 Dihydrate	Sigma-Aldrich	71643-1KG
NaCl	Carl Roth	3957.2
$(NH_4)_2SO_4$	Carl Roth	9212.1
Nunclon™ Delta Surface	Thermo Fisher Scientific	143761
Polydimethylsiloxane (PDMS)	Dow Corning	Sylgard 184
SevenExcellence Multiparameter	Mettler Toledo®	2375
Sucrose	Carl Roth	4621.2
Tubing for microfluidics	Omnilab	0.51 × 0.51 mm, Tygon®
X-Pierce™ film (flow cytometry)	Sigma-Aldrich	Z722502-100EA
2-Propanol (≥99.7 %)	VWR	20842.312
Syringe filter, Filtropur S, PES, pore size: 0.2 µm	Sarstedt	83.1826.001

Table S3: Instruments used for the described methods.

Instrument	Manufacturer/Supplier	Model/Catalog #
Amnis® CellStream® Flow Cytometer	Cytek	is100
Biopsy puncher	WPI	Reusable Biopsy Punch
Excella E24 Incubator Shaker Series	New Brunswick Scientific	SE24CM700788
Fluorescence microscope	Nikon	Eclipse Ti2 Series
Freeze-dryer	Martin Christ Gefriertrocknungsanlagen GmbH	APLHA 1-4 LDC-1M
Light engine	Lumencor	SOLA Light Engine
MicroPulser™	Bio-Rad Laboratories	1652100
Plasma generator	Diener electronic	Diener Femto Model 1 B2
Pressurized pump system	Fluigent	Microfluidic Flow Control System™
Vacuum pump	Pfeiffer Vacuums	DUO 004 B

Table S4: Nucleotide and amino acid sequence of *pHluorin2* codon-optimized for *Listeria monocytogenes* and primer used for PCR to amplify promoter P_{help} and *pHluorin2* gene.

	Sequence (DNA: 5'-to-3'; protein: N-to-C-term)	Size
gene	ATGAGTAAAGGTGAAGAATTATTTACAGGTGTTGTTCCAATTTTAGTTGAATTAGATGGTGATGTTAATG GTCATAAATTTAGTGTTAGTGGTGAAGGTGAAGGTGATGCAACATATGGTAAATTAACATTAAAATTTAT TTGTACAACAGGTAAATTACCAGTTCCATGGCCAACATTAGTTACAACATTAAGTTATGGTGTTCAATG TTTTAGTCGTTATCCAGATCATATGAAACAACATGATTTTTTTAAAAGTGCAATGCCAGAAGGTTATGTT CAAGAACGTACAATTTTTTTTAAAGATGATGGTAATTATAAAACACGTGCAGAAGTTAAATTTGAAGGTG ATACATTAGTTAATCGTATTGAATTAAAAGGTATTGATTTTAAAGAAGATGGTAATATTTTAGGTCATAAA TTAGAATATAATTATAATGAACATTTAGTTTATATTATGGCAGATAAACAAAAAAATGGTACAAAAGCAA TTTTTCAAGTTCATCATAATATTGAAGATGGTAGTGTTCAATTAGCAGATCATTATCAACAAAATACACC AATTGGTGATGGTCCAGTTTTATTACCAGATAATCATTATTTACATACACAAAGTGCATTAAGTAAAGAT CCAAATGAAAAACGTGATCATATGGTTTTATTAGAATTTGTTACAGCAGCAGGTATTACACATGGTATG GATGAATTATATAAATAG	717 bp
protein	MSKGEELFTGVVPILVELDGDVNGHKFSVSGEGEGDATYGKLTLKFICTTGKLPVPWPTLVTTLSYGVQCF SRYPDHMKQHDFFKSAMPEGYVQERTIFFKDDGNYKTRAEVKFEGDTLVNRIELKGIDFKEDGNILGHKLE YNYNEHLVYIMADKQKNGTKAIFQVHHNIEDGSVQLADHYQQNTPIGDGPVLLPDNHYLHTQSALSKDPNE KRDHMVLLEFVTAAGITHGMDELYK	238 aa
P_{help}_fw	TTTTTATATTACAGCTCCAATCATTATGCTTTGGCAGTTTATTC	
P_{help}_rv	CTTTACTCATGGGTTTCACTCTCCTTCTAC	
pHin2LM_fw	GTAGAAGGAGAGTGAAACCCATGAGTAAAGGTGAAGAATTATTTAC	
pHin2LM_rv	AGTGGTACCGCATGCCTGCACTATTTATATAATTCATCCATACCATGTG	

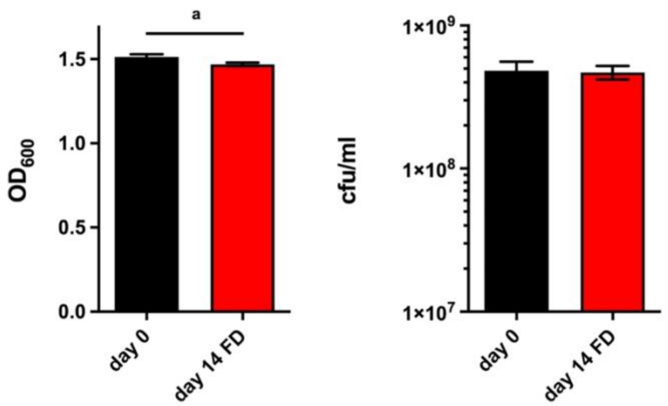

Figure S5: Effect of lyophilization and storage on *L. innocua* LMG2785/pNZ-pHin2^{Lm} biosensors in ready-to-use MTPs. OD_{600} and live bacterial counts were measured in ready-to-use MTPs immediately prior lyophilization of assay plates and after lyophilization and storage at -20°C for 14 days. All values are mean ± standard deviation of n = 3 independent cultivations of *L. innocua* LMG2785/pNZ-pHin2^{Lm}. Statistical analysis was performed by Student's t-test (unpaired, two-tailed) comparing treated vs. untreated biosensors at each timepoint. Statistically significant differences to untreated biosensor bacteria are indicated by letters (a: $p < 0.05$).
